# Harvestmen (Arachnida: Opiliones) as Overlooked Predators of Anurans in the Neotropics

**DOI:** 10.1002/ece3.73542

**Published:** 2026-04-21

**Authors:** Esteban Calvache, Osvaldo Villarreal, Cynthia Ávila‐Rojas, Alexander G. Bentley, Kenny Brito, Chiara Correa‐Zanotti, Maida Gutiérrez‐Arboleda, Katherine Iñiguez, Juan Carlos Narváez, Lizardo Proaño, Mateo Reyes‐Vizcaíno, Luís Fernando García

**Affiliations:** ^1^ Departamento de Investigación y Biología Mashpi Lodge Pacto Pichincha Ecuador; ^2^ Fundación URU Quito Ecuador; ^3^ Instituto Nacional de Biodiversidad Quito Ecuador; ^4^ Centro de Ecología Instituto Venezolano de Investigaciones Científicas (IVIC) Altos de Pipe Miranda Venezuela; ^5^ Facultad de Agronomía, Instituto y Museo del Instituto de Zoología Agrícola Universidad Central de Venezuela Maracay Aragua Venezuela; ^6^ Grupo de Investigación Ecdysis Universidad del Quindío Armenia Quindío Colombia; ^7^ Estudiante del Programa de Biología Universidad del Quindío Armenia Quindío Colombia; ^8^ Fundación Waska Amazonia Mera Pastaza Ecuador; ^9^ Red de Investigación del Corredor de Conectividad Llanganates‐Sangay Mera Pastaza Ecuador; ^10^ Centro Jambatu de Investigación y Conservación de Anfibios Fundación Jambatu Quito Pichincha Ecuador; ^11^ Departamento de expediciones Mashpi Lodge Pacto Pichincha Ecuador; ^12^ CENUR Noreste, Sede Rivera, Universidad de la República Rivera Uruguay

**Keywords:** Anurophagy, Arachnida, Neotropical food webs, trophic interactions, vertebrate predation

## Abstract

Arthropods are traditionally viewed as invertebrate prey and as predators of other invertebrates, whereas vertebrates are typically considered their predators. However, this paradigm has increasingly been challenged, particularly among arachnids. While several invertebrates are well documented as frog predators, the capacity of particular groups, such as harvestmen (Opiliones), to prey on vertebrates has remained largely anecdotal. Here we report novel field observations of anuran predation by multiple Cranaidae harvestman species across several Neotropical localities. These records include the active capture and consumption of live frogs, demonstrating their role as opportunistic mesopredators. Our findings expand current knowledge of Opiliones ecology by confirming that vertebrate predation occurs across multiple species and localities. Our results suggest that vertebrate consumption among arachnids may be more taxonomically widespread than previously recognized, highlighting the need to consider Opiliones as potential predators in Neotropical trophic networks.

## Graphical abstract

This study reports novel field observations of harvestmen (Opiliones: Cranaidae) preying on live frogs across multiple Neotropical localities, documenting active capture and consumption of anurans by these arachnids. Our findings expand the known trophic role of harvestmen as opportunistic vertebrate predators and highlight the need to consider Opiliones in Neotropical food web dynamics.

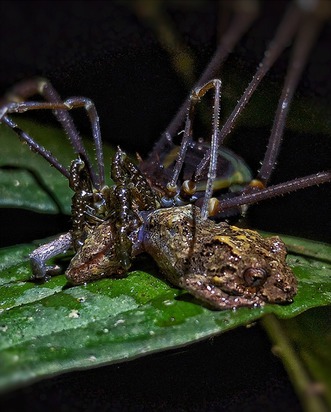

## 1 Introduction

Among terrestrial predators, arthropods frequently occupy multiple trophic positions, functioning both as intermediate predators and as top‐level carnivores in a variety of ecosystems (Garrick et al. 2019; Miller‐ter Kuile et al. 2022). For instance, spiders feed primarily on insects and other arthropods (Nyffeler and Birkhofer 2017). Because of their relatively small body size compared to vertebrates, arthropods are often portrayed mainly as prey of vertebrates, reinforcing a largely unidirectional view of trophic interactions between these groups (Mooney et al. 2010). Consequently, ecological literature has traditionally depicted arthropods as occupying intermediate positions within food webs, with comparatively little attention given to their role as predators of vertebrates (McCormick and Polis 1982).

However, this simplified perspective overlooks numerous documented cases in which arthropods prey upon vertebrates. A recent review by Valdez (2020) showed that several arthropod taxa, including insects, arachnids, centipedes, and crustaceans, are capable of preying on all major vertebrate groups. Notably, the study compiled more than 1300 records of vertebrate predation by arthropods. Although many of these observations are anecdotal, the large number of records suggests that such interactions may be far more common than previously assumed.

Among arthropods, arachnids appear to be particularly important vertebrate predators. Several arachnid orders have been reported feeding on mammals, reptiles, amphibians, and fish (Nyffeler and Altig 2020; Nyffeler and Gibbons 2021; Nyffeler and Knörnschild 2013; Nyffeler and Pusey 2014; Valdez 2020; Nyffeler et al. 2025). Spiders represent the most frequently documented arachnid predators of vertebrates, likely reflecting their diverse foraging strategies, the relatively large body size of some taxa, and their higher detectability, which increases opportunities for observation. In contrast, documented cases involving other arachnid orders remain scarce and are often fragmentary. Harvestmen (Opiliones) represent a diverse group of arachnids, with nearly 6700 described species (Kury 2013), and play important ecological roles in many terrestrial ecosystems. Although their diet may include detritus, plant material, fungi, and other resources, many species are active predators, ranging from generalists to highly specialized feeders (Acosta and Machado 2007; Nyffeler et al. 2023). Harvestmen also possess a variety of effective defenses, including a hardened body, repugnatorial secretions, autotomy, and diverse behavioral responses (Ximenes and Willemart 2024), which allow them to survive attacks from a wide range of predators, including highly aggressive groups such as ants (Villarreal et al. 2025). Although harvestmen lack venom, which in other arachnids facilitates the capture of large prey such as spiders (Foelix 2011), they are nevertheless capable of subduing prey several times their own size, including various vertebrates. Rare cases of predation on vertebrates other than amphibians have also been documented; for example, harvestmen have been reported preying on bird nestlings (Benson and Chartier 2010), highlighting the trophic flexibility of the group.

Anurans occupy key positions in Neotropical food webs, functioning both as predators and as prey (Souza, Mendonça, et al. 2023). As predators, they consume a broad range of animal taxa, including vertebrates; however, their diets consist predominantly of invertebrates, particularly arthropods (Solé and Rödder 2009). Conversely, their trophic role can also be reversed, as anurans may become prey for several arthropod groups at different life stages. For example, tadpoles are frequently attacked by aquatic insects such as odonate nymphs (Phuge et al. 2020) and by arachnids (Babangenge et al. 2019; Mamede and Nomura 2021), whereas juvenile and adult frogs may be captured and consumed by large arthropods, including insects and arachnids (Babangenge et al. 2019; Nyffeler and Altig 2020; Souza, Lisboa, et al. 2023; Valdez 2020).

Despite the growing number of reports documenting arthropods as natural enemies of anurans, available information remains limited and strongly biased toward easily observable taxa (McCormick and Polis 1982; Valdez 2020). This highlights the importance of documenting trophic interactions involving cryptic or poorly studied arthropod groups that may also play significant roles as predators of anurans.

Here, we report new records of harvestmen preying on adult anurans from multiple localities in South America. In particular, we provide evidence of predatory interactions involving several species of the harvestman family Cranaidae. Although this family has been studied in certain aspects of behavior, such as reproductive and parental care, its feeding ecology remains poorly understood. Nevertheless, available observations suggest that these harvestmen are capable of capturing and consuming live frogs, representing a noteworthy contribution given the limited knowledge of the trophic ecology of this group (Acosta and Machado 2007). Our observations support earlier reports of harvestmen preying on anurans (e.g., Castanho and Pinto‐da‐Rocha 2005; Villarreal et al. 2008; Menegucci et al. 2020) and confirm the ability of Opiliones to act as vertebrate predators. Whereas records of spider predation on frogs are relatively common and have been synthesized in several reviews (e.g., Valdez 2020), comparable interactions involving harvestmen remain rare and sporadically documented. Accordingly, this study provides new evidence of trophic interactions between harvestmen and frogs, together with an updated compilation of published records of harvestman–frog interactions. Finally, we present a global distribution map illustrating the occurrence of anurophagy in harvestmen.

## 2 Methods

Harvestmen records were compiled from original field observations and a comprehensive review of the literature. All specimens were identified from photographic records to the highest possible taxonomic level through consultation with specialists.

As no specimens were collected, prey–predator size ratios were estimated from photographs by measuring homologous body structures in pixels using ImageJ (Rueden et al. 2017). Relative measurements were obtained by comparing structures visible in the same image (e.g., body length, dorsal scute length, or prosoma length), allowing the estimation of proportional size relationships between predator and prey.

Images were analyzed using ImageJ, and measurements were repeated independently to ensure consistency. Figures were prepared in Adobe Photoshop CC 2017. The global distribution map was generated using R v. 4.5.2 (R Core Team 2025).

## 3 Results

### 3.1 Feeding Records

**FIGURE 1:**
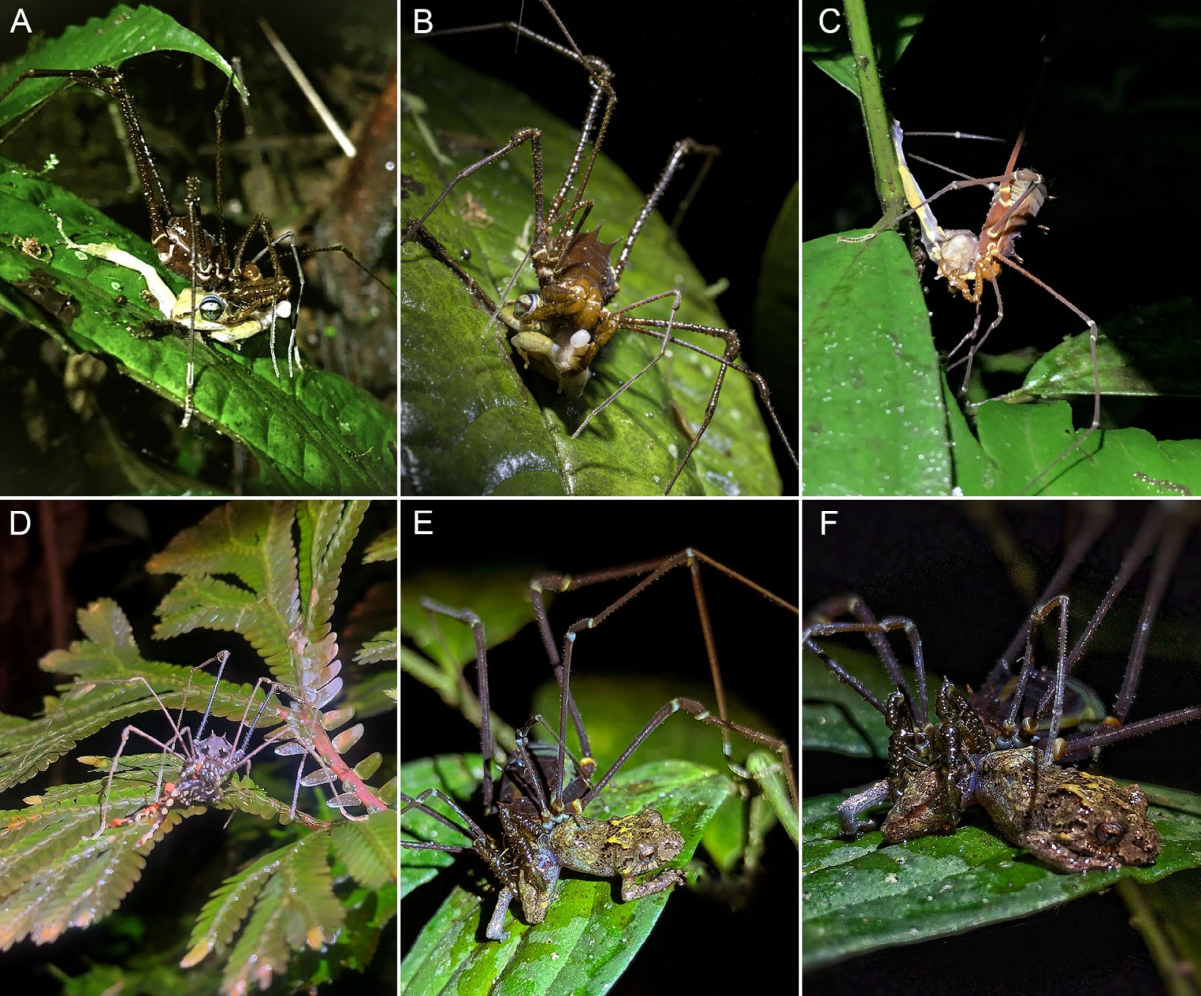
Field observations of predation on anurans by harvestmen (Cranaidae) and spiders (Araneidae). (A, B) *Holocranaus* aff. *angulus* feeding on *Pristimantis* sp. (C) *Phareicranaus lucifer* preying on the small treefrog *Dendropsophus parviceps* . (D) “*Phareicranaus*” sp. feeding on *Atelopus* sp. (E, F) *Phareicranaus* sp. consuming *Pristimantis* sp. Photos: Juan Carlos Narváez (A, B), Alexander G. Bentley (C), Katherine Iñiguez (D), Sergio Cruz (E), Maida Gutiérrez (F).

All observations of harvestmen involved adult individuals feeding on post‐metamorphic anurans. On 5 February 2020 at 20:00 h, during a field survey on the “Verrugosa” trail (0.1665°, −78.8774°, 874 m a.s.l.) in the Mashpi‐Tayra Wildlife Refuge, Ecuador, a male *Holocranaus* aff. *angulus* (Roewer 1932) was observed preying on a live *Pristimantis* sp. (Figure 1A,B). Although the specimen was not collected, precluding both species‐level confirmation and precise measurement, its size appeared comparable to that of *Holocranaus angulus* males, which Roewer (1932) originally described with a dorsal scute length of 10 mm. The harvestman held the frog by its hind limb, about 75 cm above the ground, on a leaf. Although prey capture was not recorded, the frog remained alive during partial consumption.

A similar event was recorded on 6 August 2022 at 01:15 h along the Cueva del Tigre trail (−1.4259°, −78.0361°, 1235 m a.s.l.) in the José Fiallos finca within the Ecominga Foundation's Anzu Reserve, Ecuador. A large *Phareicranaus lucifer* (Pinto‐da‐Rocha and Bonaldo 2011), approximately 8 mm in body length, was observed gripping a partially eaten clown frog, *Dendropsophus parviceps* (Boulenger, 1882) (Figure 1C), on a leaf about 80 cm above the ground, near a shallow pond above the Cueva del Tigre cave. Only half of the frog remained at the time of observation.

In another case, an individual of *Phareicranaus* sp.—conservatively identified following the possibly nonmonophyletic concept of Pinto‐da‐Rocha and Bonaldo (2011) for large Cranaidae; *Ventrivomer* sp. cannot be excluded due to image quality—was observed feeding on an *Atelopus* sp. (*spumarius*–*pulcher* complex) (Figure 1D), on 3 September 2025 at 20:15 h along the Río Napinaza (Quebrada Napinaza) in Limón Indanza, Morona Santiago Province, Ecuador (−2.9228°, −78.4089°, 1146 m a.s.l.). The event occurred on the foliage of understory vegetation approximately 150 cm above the ground near the riverbank. The harvestman was actively consuming the frog when first observed and moved away shortly afterward, leaving the body headless and partially consumed. Considering amphibian predation by harvestmen, we provide an updated list focusing on post‐metamorphic anurans (Table 1), highlighting the main families involved.

**TABLE 1:** Documented cases of anurophagy by harvestmen (Arachnida: Opiliones).

Harvestman family	Harvestman species	Anuran family	Anuran species	Date	Locality	Anuran status	References
Gonyleptidae	*Neosadocus maximus* (Giltay, 1928)	Hylidae	Metamorph of *Boana bischoffi* (Boulenger, 1887)	2001	Parque Estadual Carlos Botelho, São Paulo, Brazil	Partially consumed	Castanho and Pinto‐da‐Rocha (2005)
	*N. maximus*		Unrecognizable frog	2002		Consumed	
Cranaidae	*Phareicranaus curvipes* (Roewer, 1916)	Hemiphractidae	*Flectonotus pygmaeus* (Boettger, 1893)	Unspecified	Henry Pittier National Park, Aragua, Venezuela	Alive	Villarreal et al. (2008)
	*P. curvipes*	Eleutherodactylidae	*Eleutherodactylus* Duméril & Bibron, 1841	Unspecified		Consumed	
Gonyleptidae	*Heteromitobates discolor* (Sørensen, 1884)	Hylodidae	*Hylodes phyllodes* (Heyer & Corocoft, 1986)	2017	Private reserve in Ubatuba, São Paulo, Brazil	Alive	Menegucci et al. (2020)
	*H. discolor*		Juvenile *H. phyllodes*	2018		Dead	
Cranaidae	*Holocranaus* aff. *angulus* (Roewer, 1932)	Craugastoridae	*Pristimantis* sp.1	2020	Mashpi‐TayraWildlife Refuge, Ecuador	Alive	This study
	*Phareicranaus lucifer* (Pinto‐da‐Rocha and Kury 2011)	Hylidae	*Dendropsophus parviceps* (Boulenger, 1882)	2022	EcoMinga Foundation's Río Anzu Reserve, Pastaza Province, Ecuador	Dead	
	*Phareicranaus* sp.1	Craugastoridae	*Pristimantis* sp.2	2025	ProAves Reserve, Génova, Quindío, Colombia	Alive	
	*Phareicranaus* sp.2	Bufonidae	*Atelopus* sp. (*spumarius‐pulcher* complex)	2025	Río Napinaza (Quebrada Napinaza), Limón Indaza, Morona Santiago Province, Ecuador	Partially consumed	

Additionally, on 25 July 2025 at 21:00 h in the village of Pedregales Alto, Génova, Quindío, Colombia (4.173047° N, −75.734339° W; 2100 m a.s.l.), an individual of the harvestman *Phareicranaus* sp. was observed preying on a *Pristimantis* sp. (Figure 1E,F; Video S1). The harvestman held the live frog by its right hind limb using chelicerae and pedipalps. No resistance was observed from the frog while it was being consumed. The harvestman was similar in size to the frog (prey: predator size ratio = 1.29), although exact measurements could not be obtained.

## 4 Discussion

Although harvestmen lack venom, unlike most other arachnid orders, which would seemingly limit their ability to subdue large and mobile prey, active predation on postmetamorphic anurans has remained poorly documented and largely anecdotal. Our observations, which include multiple predation events involving several harvestman species and different frog taxa, suggest that such interactions may occur more frequently than previously recognized. Traditionally regarded as broad‐spectrum omnivores that consume plant material, fungi, vertebrate feces, insects, and conspecifics (Acosta and Machado 2007), harvestmen are nevertheless capable of actively preying on small vertebrates. Within this broad trophic niche, predation on vertebrates such as anurans appears to represent an uncommon but ecologically meaningful behavior (Villarreal et al. 2008; Menegucci et al. 2020).

Several morphological traits may facilitate the capture of vertebrate prey in large‐bodied harvestmen. Many Laniatores possess a heavily sclerotized dorsal scutum, robust chelicerae, and spined pedipalps that may assist in restraining struggling prey (Shultz and Pinto‐da‐Rocha 2007). In addition to aiding prey capture, heavy armature may also reduce the risk of predation during such interactions. Experimental evidence indicates that strongly armored harvestmen can effectively resist attacks from potential predators, suggesting that body armature functions as an important defensive mechanism (da Silva Souza and Willemart 2011). More broadly, defensive behavior and survivorship during interactions with aggressive predators such as ants may further reduce the costs of risky foraging or predatory attempts (Villarreal et al. 2025). Consequently, large‐bodied and heavily armored species may be able to attempt attacks on relatively large prey, such as small frogs, with comparatively low risk.

Sexual dimorphism may also influence predatory performance in some harvestmen. Many species exhibit pronounced morphological differences between males and females, particularly in traits such as body size, chelicerae, and pedipalps, which are frequently associated with male–male competition, territorial behavior, and reproductive interactions (Machado and Macías‐Ordóñez 2007; Shultz and Pinto‐da‐Rocha 2007). Because these same structures are also involved in prey capture and manipulation, sexually dimorphic morphology may influence prey‐handling ability and potentially affect hunting performance in certain species. However, only two of the four individuals observed in our records could be reliably identified as males from photographs, while the remaining two could not be sexed. This limited sample prevents a robust evaluation of whether predation events were associated with a particular sex. Future studies should therefore assess whether sexually dimorphic morphology influences prey capture or prey choice in harvestmen. Although not all harvestmen species were observed capturing live prey, and scavenging cannot be ruled out in some cases given their omnivorous habits, our records support previous evidence that certain Opiliones are capable of subduing relatively large vertebrates. In our observations, prey size was comparable to that of the predator, consistent with patterns reported for other arthropod predators of frogs, such as carabid beetles of the genus Epomis (Wizen and Gasith 2011) and several spider taxa (Meneses et al. 2020). The relatively soft integument of amphibians may facilitate this type of predation (Valdez 2020). Nevertheless, the ability of these typically slow‐moving arachnids to capture evasive vertebrates raises important questions regarding their predatory strategies. Harvestmen may rely on ambush behavior, mechanical immobilization using their pedipalps and chelicerae, or positional advantages when frogs climb vegetation. Detailed behavioral observations will therefore be necessary to better understand how harvestmen overcome the mobility and escape responses of amphibian prey.

**FIGURE 2:**
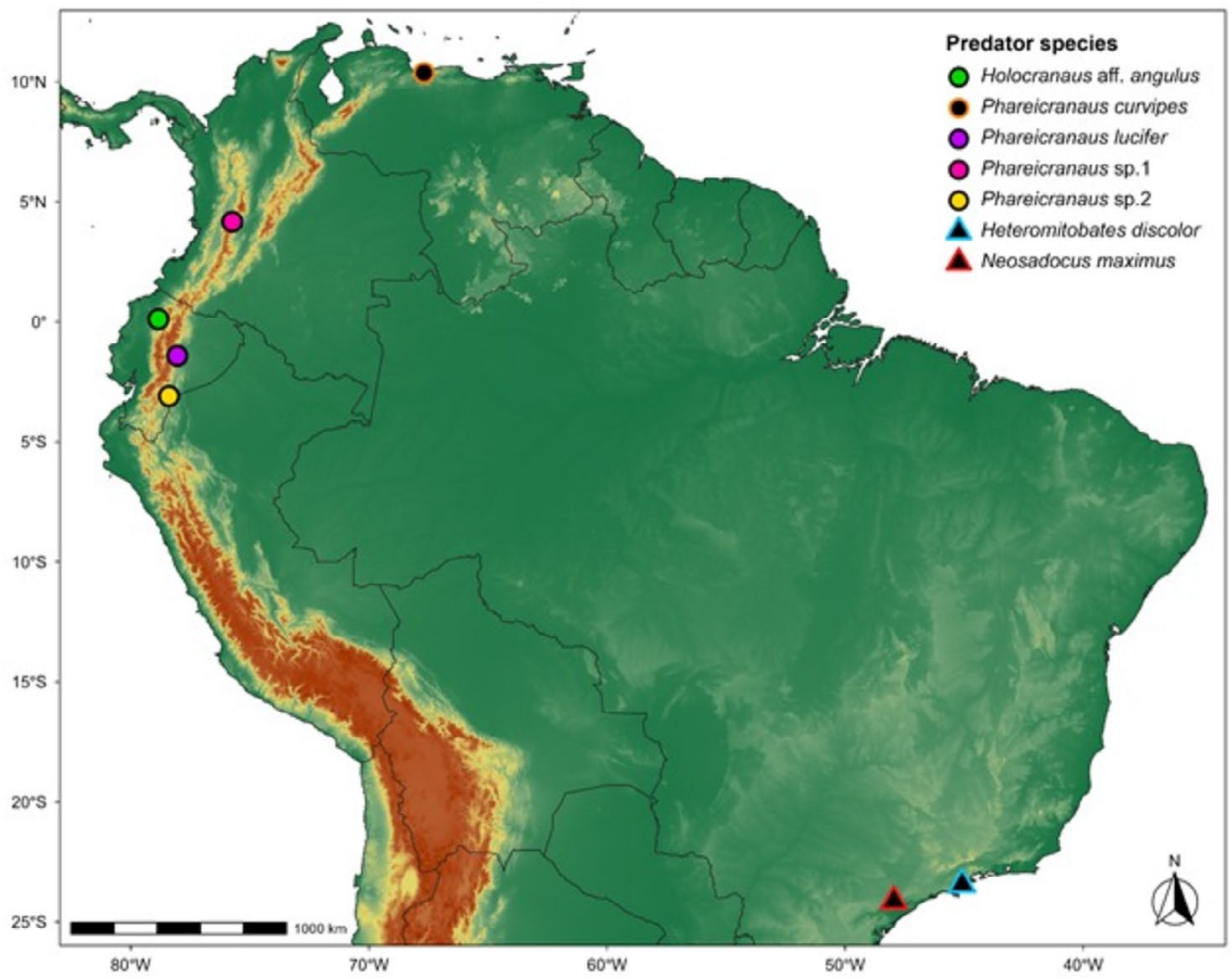
Global distribution of recorded anurophagy (predation on anurans) by harvestmen (Opiliones). Circles represent predation events by species of the family Cranaidae, and triangles represent events by the family Gonyleptidae. Black‐filled symbols with colored outlines indicate records compiled from published literature, whereas colored symbols indicate newly documented records from this study.

Interestingly, the predation events compiled here involve two distinct harvestman lineages from different South American biomes: Cranaidae in the Andes and Gonyleptidae (Gonyleptinae) in the Atlantic Forest (Figure 2). This parallel echoes recent discussions on ecological equivalence between cranaids and goniosomatine gonyleptids, which show convergent morphologies. Although the gonyleptid predators in our records belong to Gonyleptinae rather than Goniosomatinae, the emergence of vertebrate predation in these distantly related, large‐bodied lineages raises the question of whether this behavior represents another dimension of ecological convergence or simply reflects the capacity of heavily armored forms to exploit vertebrate prey when opportunities arise.

Beyond representing a substantial biomass resource, vertebrate prey may also provide important nutritional benefits. While some vertebrates have been suggested to constitute relatively low‐quality prey for spiders (Wilder and Simpson 2022), other studies indicate that amphibians can provide essential micronutrients that positively affect spider fitness (Hunsucker et al. 2025). Given the physiological and digestive similarities between harvestmen and spiders, it is plausible that frogs may confer comparable nutritional advantages when consumed by harvestmen. Such benefits may be particularly relevant in species with energetically demanding behaviors. For example, males in some cranaid species exhibit egg‐guarding behavior and may remain with the clutch for extended periods, limiting their opportunities to forage (Sewlal and Hook 2014). Under these circumstances, the occasional consumption of relatively large prey items such as vertebrates could represent an important energetic resource. In light of the growing recognition of micronutrients as key drivers of food web dynamics (Kaspari 2021), understanding vertebrate predation in arachnids may reveal overlooked pathways of nutrient transfer and help refine current assumptions regarding their trophic position. Future studies should investigate vertebrate predation across other arachnid groups and assess whether arthropods exhibit selectivity toward particular vertebrate prey. Moreover, given that some of the frogs involved in these interactions correspond to undescribed species, our findings also highlight the importance of documenting the still poorly known diversity of Neotropical fauna and formally describing these species to support conservation efforts.

## Author Contributions

**Esteban Calvache:** conceptualization (supporting), data curation (equal), funding acquisition (equal), investigation (supporting), methodology (equal), project administration (equal), resources (equal), validation (equal), writing – original draft (equal), writing – review and editing (equal). **Osvaldo Villarreal:** conceptualization (lead), data curation (equal), investigation (lead), methodology (equal), supervision (equal), validation (equal), visualization (lead), writing – review and editing (lead). **Cynthia Ávila‐Rojas:** data curation (supporting), investigation (supporting), resources (supporting), visualization (equal), writing – review and editing (equal). **Alexander G. Bentley:** conceptualization (supporting), data curation (equal), funding acquisition (equal), investigation (supporting), resources (equal), writing – review and editing (equal). **Kenny Brito:** conceptualization (supporting), data curation (equal), investigation (supporting), resources (equal), writing – review and editing (supporting). **Chiara Correa‐Zanotti:** conceptualization (supporting), data curation (equal), investigation (supporting), methodology (equal), writing – review and editing (equal). **Maida Gutiérrez‐Arboleda:** data curation (equal), resources (equal), writing – review and editing (equal). **Katherine Iñiguez:** data curation (equal), investigation (supporting), methodology (equal), resources (equal), writing – review and editing (equal). **Juan Carlos Narváez:** conceptualization (supporting), data curation (equal), methodology (equal), resources (equal), writing – review and editing (equal). **Lizardo Proaño:** data curation (equal), investigation (supporting), resources (equal), writing – review and editing (equal). **Mateo Reyes‐Vizcaíno:** data curation (equal), investigation (supporting), methodology (equal), resources (equal), software (equal), visualization (lead), writing – review and editing (equal). **Luís Fernando García:** conceptualization (lead), data curation (equal), investigation (lead), methodology (equal), writing – review and editing (lead).

## Funding

The authors have nothing to report. Fieldwork and logistical support were provided by the Mashpi Lodge, Ecominga Foundation, Fundación ProAves de Colombia, and Re:wild as acknowledged above.

## Conflicts of Interest

The authors declare no conflicts of interest.

## Supporting information


**Video S1:** Predation of *Pristimantis* sp. by the harvestman *Phareicranaus* sp. in Pedregales Alto, Quindío, Colombia.

## Data Availability

All data supporting this study are included within the article and its Supporting Information. The photographic records of predation events are available from the corresponding authors upon reasonable request. No new datasets were generated or analyzed during this study.
